# The unacceptable situation of opportunistic screening for breast cancer in Brazil

**DOI:** 10.1590/1806-9282.20240651

**Published:** 2024-09-16

**Authors:** Aline Ferreira Bandeira de Melo Rocha, Ruffo Freitas, Leonardo Ribeiro Soares, Luciano Fernandes Chala

**Affiliations:** 1 Universidade Federal de Goiás, School of Medicine – Goiânia (GO), Brazil.; 2 Universidade Federal de Goiás, Teaching Hospital, Mastology Program – Goiânia (GO), Brazil.; 3 Araújo Jorge Hospital, Goiás Anticancer Association – Goiânia (GO), Brazil.; 4 Fleury Medicine and Health Group – São Paulo (SP), Brazil.; 5 Brazilian College of Radiology – São Paulo (SP), Brazil.

Dear Editor,

In a recent article published in *PLoS One* ^1^, the authors reached conclusions concerning breast cancer screening in Brazil in women aged 40–49 years that strongly contradict actual data. This letter defends the importance of screening in this age group based on actual nationwide data and statistics.

That study^1^ assumed an attendance rate for breast cancer screening of 80%, which is much higher than the actual rate registered for Brazil. In fact, the mean breast cancer screening rate for the 50–69-year-old age group within the public healthcare system (SUS) between 2013 and 2019 was as low as 36.71%, with a significant drop occurring in recent years due to the COVID-19 pandemic^2^. Consequently, the coverage rate for the 40–49-year-old age group is expected to be even lower since screening is not endorsed for these women within the SUS^3^.

Analysis of the clinical stages of breast cancer within the SUS^4^ showed an increase in the rates of advanced stages (III and IV) at diagnosis in both age groups (40–49 and 50–69 years), with the prevalence being greater in the younger group, as shown in Table 1. In the private healthcare system in Brazil, in which women aged 40–49 years are screened, the prevalence rates of stages III and IV reported in the 2018–2021 period ranged from 34.5 to 36.6% in women under 50 years of age^5^.

**Table 1:** Breast cancer stages III and IV as detected at diagnosis in women seen in the public healthcare system (SUS) according to age group, between 2013 and 2022.

Age group	2013	2014	2015	2016	2017	2018	2019	2020	2021	2022
40–49 years, n (%)	3,458 (50)	3,233 (50)	3,177 (53)	3,398 (53)	3,589 (54)	3,682 (54)	4,106 (54)	4,071 (56)	4,714 (58)	5,069 (59)
50–69 years, n (%)	6,234 (45)	5,879 (46)	6,016 (47)	6,354 (47)	6,634 (47)	7,347 (48)	7,824 (48)	7,891 (52)	9,265 (52)	9,929 (52)

Although in the private healthcare system, the rate of advanced stages detected in women under 50 years of age is lower than that in the SUS, the overall issue remains concerning and highlights a need to initiate screening at 40 years of age. This initiative will enable early detection, timely treatment of the disease, and a desirable and necessary reduction in mortality^6^.

The *PLoS One* study^1^ presents data that are not in line with current statistics on the incidence and mortality of breast cancer in low- and middle-income countries. Most breast cancer cases in the world are found in these countries, with an incidence rate of 62.3% and a mortality rate of 79.3% in women aged 50–69 years and an incidence rate of 71.8% and a mortality rate of 86.8% in those aged 40–49 years ^7^. These data highlight the importance of implementing breast cancer screening in these countries to enable early detection and increase the likelihood of a cure.

Therefore, we question why breast cancer screening is still not recommended for women aged 40–49 years, since screening clearly allows cancer cases to be detected at an early stage, which is crucial for the success of treatment and the quality of patients’ lives. In view of the evidence presented, it is vital to support increasing the access of women aged 40–49 years to breast cancer screening. This requires implementing public policies that guarantee women the right to undergo screening within the SUS, make them aware of the importance of early detection, and combat disinformation.
